# Comforting styles of serious illness conversations: a Swiss wide factorial survey study

**DOI:** 10.1186/s12916-025-04046-6

**Published:** 2025-04-14

**Authors:** Robert Staeck, Carsten Sauer, Steven M. Asch, Sofia C. Zambrano

**Affiliations:** 1 Institute of Social and Preventive Medicine (ISPM), University of Bern, Bern, Switzerland; 2 Graduate School for Health Sciences, University of Bern, Bern, Switzerland; 3 Faculty of Sociology, University of Bielefeld, Bielefeld, Germany; 4 Department of Medicine, Stanford University School of Medicine, Stanford, CA USA

**Keywords:** Communication, End-of-life, Comfort, Physician–patient relationship

## Abstract

**Background:**

Serious illness conversations can cause discomfort in patients, potentially impeding their understanding and decision-making. Identifying ways in which physicians can reduce this discomfort may improve care. This study investigates which physician communication styles and characteristics individuals perceive as comforting in physician–patient serious illness conversations.

**Methods:**

We conducted a nationwide online factorial survey in German, French, and Italian with 1572 Swiss participants from the public (51.4% women) aged 16 to 94. Each participant assessed 5 out of 1000 different vignettes describing a physician informing a cancer patient about their terminal prognosis. We systematically manipulated 11 attributes: physician’s years of experience, physician sex, patient sex, patient age, prior relationship to physician, clarity of information, self-disclosure, physician taking time, recommendation, expression of sadness, and continuity of care. Participants evaluated their comfort level with the physician described in the vignettes. Multilevel models with random effects were used to analyze the impact of the dimensions on comfort.

**Results:**

Clarity of information (*β* = 2.13, *p* < 0.01), taking enough time (*β* = 2.00, *p* < 0.01), and continuity of care (*β* = 1.27, *p* < 0.01) were the strongest predictors of comfort. A prior physician–patient relationship significantly increased comfort, with a longer relationship being more comforting (*p* < 0.01). Physician self-disclosure (*β* = 0.40, *p* < 0.01) and expression of sadness (*β* = 0.46, *p* < 0.01; *β* = 0.58, *p* < 0.01) also increased comfort. Recommendations based on experience did not influence comfort but failing to provide reasons for recommendations decreased comfort (*β* = − 0.24, *p* < 0.01). Recommendations based on patient preference increased comfort (*β* = 0.30, *p* < 0.01). A limitation of this study is that the vignettes describe only fictitious situations and can thus be seen as oversimplifications.

**Conclusions:**

Taking time, providing clear information, and ensuring continuity of care are pivotal in enhancing comfort. Also relevant are the expression of sadness, physician self-disclosure, and a prior relationship with the patient.

**Supplementary Information:**

The online version contains supplementary material available at 10.1186/s12916-025-04046-6.

## Background

Serious illness conversations (SICs) refer to discussions between healthcare providers, patients, and their families focused on patients’ values, prognosis, treatment options, and goals of care related to serious or life-threatening illnesses [1, 2]. These conversations are crucial yet often emotionally fraught for both physicians and patients [1, 3]. Physicians report fearing using the wrong words and concern about managing the patients’ response and emotions [4]. However, engaging patients in discussions about their values and goals strengthens trust and ensures that they feel supported and respected [5]. SICs can give patients a sense of control during uncertain times in their illness, fostering awareness and understanding of the disease and prognosis [6]. SICs help patients maintain hope while acknowledging the reality of advanced illness or even death [7].

How physicians conduct SICs can promote patient comfort and facilitate effective communication. Prior research has tested comforting language [2, 8] and has found that communication style can reduce fear and anxiety and improve quality of life and recall of prognostic information [1, 9]. What styles are most effective in producing these outcomes has been less studied [10]. A video-vignette study showed that incorporating affect into communication was effective during bad news consultations in reducing anxiety and improving recall [11]. Westendorp et al. [12] showed that clinicians’ behaviors like not interrupting the patient, adopting an empathic tone of voice, and empathic responses to the patient-expressed emotions also increased information recall in patients with advanced breast cancer [12]. Empirical evidence supports that the majority of patients with advanced cancer want a physician who listens to their distress and concerns [13] and want to feel heard and listened to [14]. Communication styles that include dialogic information exchanges such as shared decision-making also have the potential to help regulate patients’ emotions and facilitate comprehension of medical information [15, 16].

Characteristics of the physician–patient relationship have also been found to play an important role in patient comfort during SICs. Hillen et al. [17] highlight that trusting physician–patient relationships can facilitate communication and medical decision-making, decrease patient fear, and improve treatment adherence. A previous study on patients’ relationships with their palliative care physicians found that patients desired a more personal connection and patients expect characteristics like honesty, good listening skills, taking time, experience, gentleness, and knowledge of the patient’s history [18, 19]. Having a long personal relationship with the clinician (oncologists, nurses, or allied health) was found to foster a sense of comfort and trust [20] and empower the patient to engage in discussions more actively [21, 22]. Studies have shown that ruptures of continuity can lead to feelings of abandonment and potentially leave patients with the feeling that the physician did not care [19, 23, 24]. Additionally, explicit prognostic information and reassurance about no abandonment when entering palliative care were found to decrease participants’ uncertainty and to increase their self-efficacy and satisfaction [25].

Physician self-disclosure of personal information or experiences can also be used to strengthen the physician–patient relationship [26] and has been found to increase comfort and satisfaction [27] in addition to fostering an atmosphere that encourages patients to feel more comfortable sharing [28]. However, self-disclosure has been described as a boundary violation [29]. Studies have reported decreased comfort and satisfaction when primary care physicians self-disclose [27]. While these inconsistent findings may be attributable to variations in the initial nature of the physician–patient relationship, the context of self-disclosure, and the specific content of the self-disclosure [28], physician self-disclosure may be a skill that could improve patient comfort during SICs.

Despite the known importance of specific communication styles and characteristics, there is a need for a deeper understanding of how they may contribute to comfort during SICs. Previous studies in the field have predominantly relied on observational methods and thus may be confounded. Factorial surveys offer a solution by combining useful elements of experiments and surveys, allowing for the assessment of how single attributes affect variables/outcomes of interest. Many attributes can be tested simultaneously, while their effects can be assessed independently [30]. In this study, through the systematic manipulation of variables in scenarios (e.g., physician communication styles and relationship characteristics), factorial designs allow insight into underlying mechanisms contributing to comfort with serious illness conversations. Additionally, factorial designs facilitate the evaluation of multiple scenarios by one participant, even those that rarely occur, leading to higher number of rated scenarios and thus increasing statistical power. Furthermore, most studies are single-institutional, highlighting the need for national surveys to enhance generalizability and representativeness. Research in healthcare communication has established that members from the general public can be seen as “analogue patients,” that is, participants who are asked to imagine themselves being in a patient’s role. This approach has been shown to be valid for studying communication in healthcare and can reliably reflect patient perspectives [31–33], and has been used as proxies for patients in a vignette study [34]. By employing this method, our study contributes unique insights into both public perceptions of SIC, which are relevant for understanding societal attitudes toward such discussions, and how individuals, when adopting a patient perspective, may respond to these discussions. Therefore, we aimed to examine how different physician communication styles and relationship characteristics contribute to comfort during SICs using an experimental factorial design.

## Methods

### Study design

We conducted a cross-sectional experimental factorial survey [35–37] in which we systematically manipulated 11 dimensions with a focus on physician characteristics and communication styles during a SIC but also including patient age and gender to systematically control for them. The study was assessed by the Bernese Cantonal Research Ethics Committee, which determined that the study did not require a full ethics application (BASEC-Nr. Req- 2022–01349) thus not requiring written informed consent. This study was part of a larger study encompassing the dependent variables: comfort, trust, professionalism, compassion, empathy, willingness to follow a physician’s recommendation, and end-of-life specificity. This study was not including patients or the public during development.

### Study participants

Participants were recruited in collaboration with gfs.bern, an opinion research company specializing in representative surveys and data analyses. We employed quotas for age, gender, and language (German, French, and Italian are national languages of Switzerland) to ensure a representative sample in these areas. Potential participants were contacted by e-mail with a link to the survey using gfs.bern online convenience panel. The link was configured by gfs.bern in such a way that participants were only able to participate once. Recruitment lasted from April 27 to June 3, 2023. Informed consent to participate was given on the first page of the online survey. Due to the nature of quota sampling, it is not feasible to calculate a response rate.

### Instrument

The full-factorial design combining all possible levels comprises 55'296 scenarios or vignettes (= 3 × 2 × 2 × 3 × 4 × 2 × 2 × 2 × 6 × 4 × 2). Out of this full factorial, we drew 1000 vignettes. We describe this process in detail in the “Statistical analysis” section. The vignettes were included in the online survey, which also contained 22 sociodemographic questions. Translation of the vignettes from English to German, French, and Italian was performed by native speakers using forward and backward translation. The survey was piloted using a convenience sample (*N* = 15), ensuring that German, Italian, and French-speaking people provided feedback. We set up the online survey in Qualtrics.

### Dependent and independent measures

The dependent measure in this study was the participants’ comfort with the physician depicted in the vignette which was assessed using the question: “If you were the patient, how comfortable would you feel with this physician.” We measured comfort on an 11-point rating scale ranging from − 5 to + 5 with verbal anchors for the minimum score (− 5, not at all) and maximum (+ 5, totally), following recent recommendations. Vignette dimensions and their levels were the independent measures based on a previous literature review and are presented in Table 1. The selected dimensions encompassed both factors previously demonstrated to influence SIC and exploratory, emotionally relevant dimensions that could potentially impact SIC. In total, the following 11 dimensions were investigated. *Experience of the physician* was categorized into three levels (early, mid, and late career) to represent key stages of a medical career and allow for the assessment of potential non-linear relationships between experience and comfort. *Sex of physician* [38] and *sex of patient* [38] as varied between two categories (female and male). *Patient age* was also divided into three groups to explore differences among young, middle-aged, and older patients. *Prior relationship to physician* [20] had three levels (no relationship, short-term, or long-term) to examine its influence on comfort. *Clarity of information* [25] was tested with two levels to assess its role in perceived comfort. Similarly, *self-disclosure* [26, 38] and *physician takes time* [18] were each examined with two levels to evaluate their impact on comfort ratings. *Recommendation* [2, 39] had six levels, balancing the absence of recommendations against those provided based on patient preferences, physician experience, or without a stated reason, to determine whether the way a recommendation is given affects comfort. *Expression of sadness* was varied across three levels to analyze both its influence on comfort and whether the manner of expression plays a role. Lastly *continuity of care* [40, 25] was either present or absent, allowing for an assessment of its effect on comfort. An example of a complete vignette can be found in Additional file 1: Table S1.

**Table 1:** Vignette dimensions and their levels

Dimensions	Levels	Textual
Experience of physician	Early career Mid career Late career	1. 5 years of experience 2. 15 years of experience 3. Over 25 years of experience
Sex of physician	Male Female	
Sex of patient	Male Female	
Age of patient	Young adult Middle aged adult Old adult	1. 35 years old 2. 55 years old 3. 80 years old
Prior relationship to physician	No relationship No relationship Short relationship Long relationship	1. Is meeting the physician for the first time 2. Is meeting the physician for the first time 3. Has known the physician since this recent hospitalization 4. Has known the physician since the beginning of the illness
Clarity of information	Brief and technical Detailed and understandable	1. Only brief explanations of the disease and prognosis with complicated and technical language that the patient finds hard to understand 2. Detailed and clear explanations of the disease and prognosis that the patient seems to understand well
Self-disclosure	No Yes	1. (blank) 2. The physician tells the patient that his/her father had the same illness and understands how difficult the situation is
Physician takes time	No Yes	1. Little to no time to listen to the patient or to answer questions 2. Enough time to listen to the patient and to answer questions
Recommendation	No No No Yes, without a reason Yes, based on experience Yes, based on patient preference	1. Based on the information given, the patient needs to decide by himself/herself if chemotherapy should be continued 2. Based on the information given, the patient needs to decide by himself/herself if chemotherapy should be continued 3. Based on the information given, the patient needs to decide by himself/herself if chemotherapy should be continued 4. They should stop chemotherapy 5. Based on their experience, their recommendation is to stop chemotherapy 6. Based on the wishes expressed by the patient during the conversation, their recommendation is to stop chemotherapy
Expression of sadness	No No Yes, in words Yes, in words + tearing up	1. (blank) 2. (blank) 3. The physician expresses how sad it makes him/her to give these bad news 4. The physician expresses how sad it makes him/her to give the bad news and tears up
Continuity of care	No Yes	1. He/she will lose contact with the patient, because they will be referred to a different team of physicians 2. He/she will remain available for the patient, despite the fact that a new team of physicians will now be involved in their care

Prior relationship to physician, recommendation, and expression of sadness are balanced, which means they reflect equal representation of “no relationship” and “relationship” (short and long); (blank) indicates that those sections were not displayed in the vignettes

### Statistical analysis

**Fig. 1:**
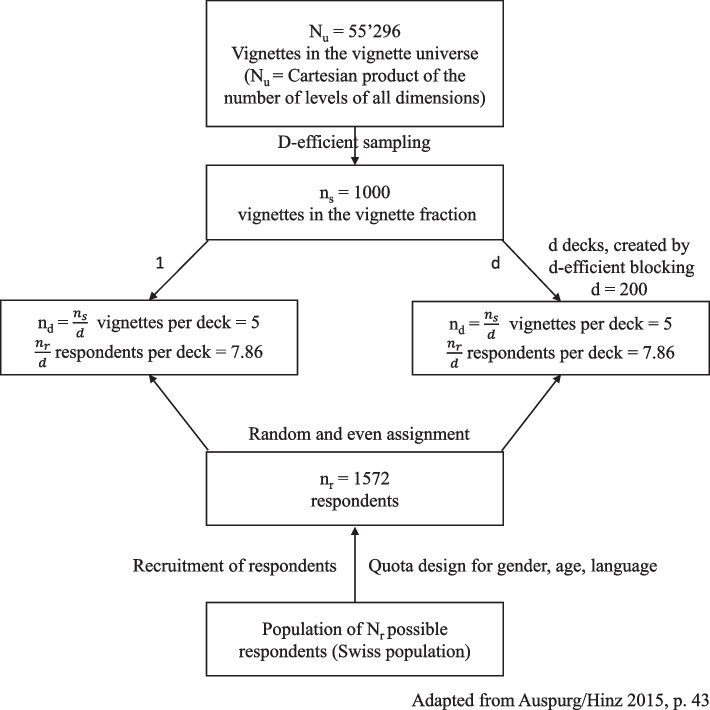
Overview of vignette universe, deck size, and respondent assignment

Out of all possible scenarios (vignette universe = 55'296), a fractionalized experimental design (D-efficient design) of 1000 vignettes was drawn using SAS (D-efficiency = 98.87). D-efficient designs are characterized by sampling vignettes with a minimal intercorrelation of dimensions (and interaction terms) and a maximal variance of vignette levels [41]. Correlation of dimensions can be found in Additional file 1: Table S2. This vignette sample was then blocked into 200 decks of 5 vignettes and randomly and evenly assigned to participants, without randomizing the order of the vignettes. We aimed for a minimum of five participants per vignette. Figure 1 provides an overview of the size of the vignette universe, deck size, and respondent assignment.

The factorial survey data have a multi-level structure as each respondent evaluated five vignettes. To address this specific data structure, we applied multilevel models with random effects using STATA (version 16.1) [42]. We estimated and report the main effects for the vignette dimensions. Reference categories for categorical dimensions were *early career*, *no prior relationship with physician*, *35 year old patient*, *brief and technical information*, *no self-disclosure*, *having only limited time*, *no recommendation*, *no expression of sadness*, and *no continuity of care*. Coefficients indicate the increase or decrease on the 11-point rating scale for comfort scores, with all other factors remaining unchanged. Coding of the dimensions and levels can be found in Additional file 1: Table S3. Missing values were listwise deleted for analysis. For subgroup analyses, subgroups were built based on existing participant categories of interest: male vs. female, worked in healthcare vs. not, chronic disease of self or someone close vs. not. The significance level in this study was alpha < 0.05.

## Results

### Respondent characteristics

A total of 1572 (51.4% female) participated in the study. On average, participants were 55.6 years old (SD = 17.64). Over 90% of participants had Swiss citizenship (91.3%), and the majority was German speaking (65.3%). Most participants had attended some form of higher education (60.1%). Table 2 shows all assessed sociodemographic variables.

**Table 2:** Respondent characteristics

Variable	Participants, *n* (%)
Gender	
Female	808 (51.4)
Male	756 (48.1)
Age, M (SD)	50.6 (17.64)
Primary language	
German	1027 (65.3)
French	429 (27.3)
Italian	75 (4.8)
Other	41 (2.6)
Nationality	
Swiss	1435 (91.3)
Other	136 (8.7)
Religion	
Protestant Reformed	422 (26.8)
Roman Catholic	405 (25.8)
Muslim and Islamic communities	9 (0.6)
Jewish religious communities	5 (0.3)
Other churches and religious communities	46 (2.9)
No religious affiliation	682 (43.4)
Education	
No school qualification	2 (0.1)
Mandatory school	28 (1.8)
Vocational training	319 (20.3)
Matura	255 (16.2)
Higher education	946 (60.1)
Other	21 (1.3)
Worked in healthcare, yes	324 (20.6)
Physician	29 (1.84)
Nurse	101 (6.42)
Other medical personnel	92 (5.85)
Other	102 (6.49)
Chronic disease of self or someone close	529 (33.65)

Note. Worked in healthcare others are for example administrative personnel, IT, or consulting

The average completion time without participants who took longer than an hour (*N* = 68, system time kept running when people closed the survey and reopened it later) was 13.7 min.

### Vignette characteristics

**Fig. 2:**
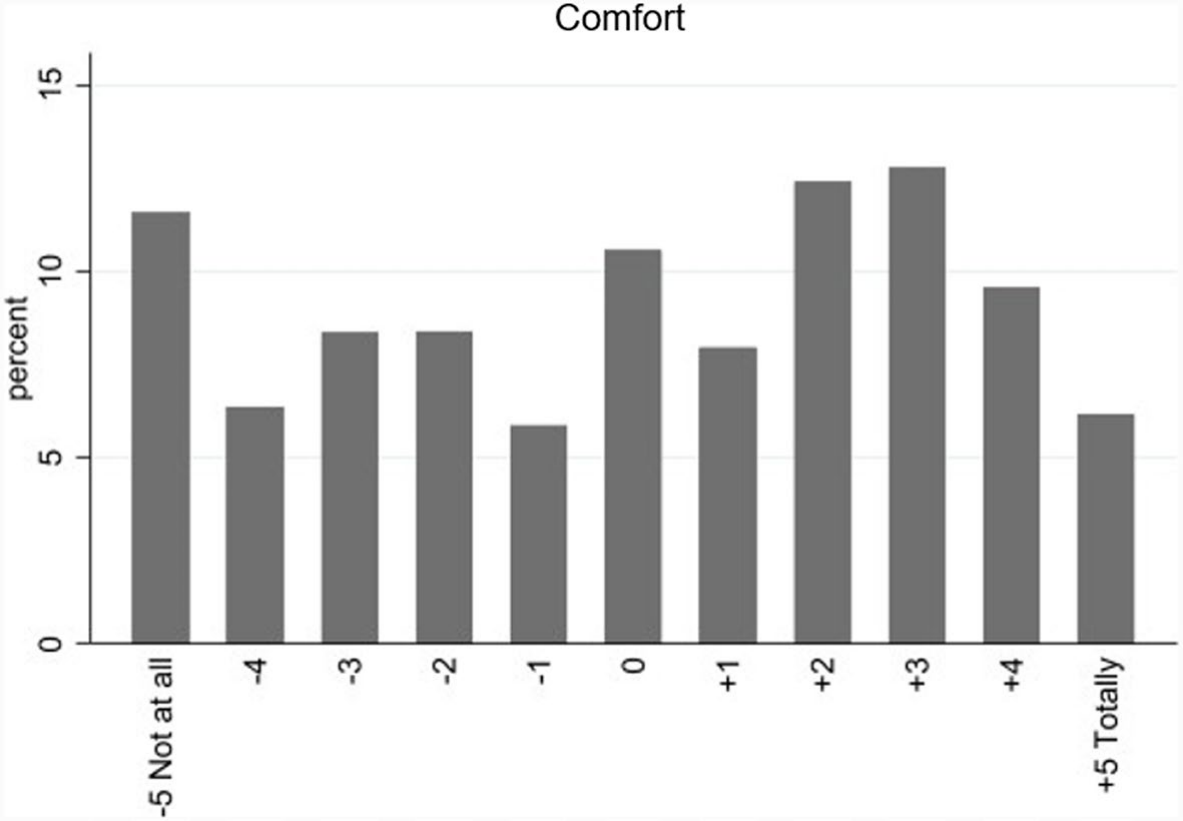
Distribution of comfort ratings across all vignettes

Each deck (consisting of five different vignettes) was rated at a minimum by 5 different participants and at a maximum by 10 different participants, equaling to 7860 vignettes rated in total. For 103 vignettes (1.31%), no comfort rating was given. Figure 2 shows the distribution of comfort across all vignettes.

### Prediction of comfort

Our model explained 27.3% of the variance, and comfort was significantly predicted by the 11 dimensions (Wald Chi^2^_(17)_ = 4257.36; *p* < 0.05). *Clarity of information delivery* (*β* = 2.13, *p* < 0.01), *taking enough time* (*β* = 2.00, *p* < 0.01), and mentioning *continuity of care* (*β* = 1.27, *p* < 0.01) were the strongest predictors of comfort in the model. A *prior relationship* (short: *β* = 0.26, *p* < 0.01; long: *β* = 0.44, *p* < 0.01) with the physician enhanced comfort, and a *longer relationship* contributed even greater to comfort (*p* < 0.01). Physician *self-disclosure* (*β* = 0.40, *p* < 0.01) and *physician expression of emotion* (*β* = 0.46, *p* < 0.01; *β* = 0.58, *p* < 0.01) were also found to increase participants’ comfort ratings. However, no difference was found between *expressing sadness in words* compared to *expressing it in words and tearing up* (*p* = 0.10). *Failing to provide reasons for recommendations*, which the physician gave, decreased comfort (*β* = − 0.25, *p* < 0.01). *Physician’s recommendations based on experience* did not influence comfort but if *based on patient preference*, increased it (*β* = 0.30, *p* < 0.01). All main effects of the investigated dimensions can be found in Table 3.

**Table 3:** Main effects of the vignette dimensions on comfort

Comfort	*β*	Std. err	*Z*	*P* >|*z*|	95% conf. interval	
Experience						
Early career	Ref	Ref	Ref	Ref	Ref	Ref
Mid career	0.151	0.062	2.420	0.016	0.029	0.273
Late career	0.268	0.062	4.320	> 0.001	0.147	0.390
Sex physician (male)	− 0.088	0.051	− 1.730	0.083	− 0.188	0.012
Sex patient (male)	0.008	0.051	0.160	0.867	− 0.091	0.108
Age patient						
30	Ref	Ref	Ref	Ref	Ref	Ref
55	− 0.263	0.621	− 0.42	0.672	− 0.148	0.955
80	0.163	0.062	2.62	0.009	0.041	0.285
Prior relationship						
No	Ref	Ref	Ref	Ref	Ref	Ref
Short	0.256	0.062	4.110	> 0.001	0.134	0.378
Long	0.444	0.062	7.150	> 0.001	0.323	0.566
Clarity of information (yes)	2.134	0.051	42.000	> 0.001	2.035	2.235
Self-disclosure (yes)	0.404	0.051	7.960	> 0.001	0.305	0.504
Time (long)	1.998	0.051	39.290	> 0.001	1.898	2.098
Recommendation						
No	Ref	Ref	Ref	Ref	Ref	Ref
Yes, without reason	− 0.245	0.072	− 3.410	0.001	− 0.386	− 0.104
Yes, based on experience	− 0.017	0.072	− 0.240	0.809	− 0.158	0.124
Yes, patient based on patient preference	0.301	0.072	4.210	> 0.001	0.161	0.442
Expression of sadness						
No	Ref	Ref	Ref	Ref	Ref	Ref
Yes, in words	0.458	0.062	7.370	> 0.001	0.336	0.580
Yes, in words and tears up	0.577	0.062	9.250	> 0.001	0.455	0.699
Continuity of care	1.266	0.051	24.900	> 0.001	1.166	1.366
Constant	2.611	1.018	25.650	> 0.001	2.411	2.810

To illustrate the range of comfort ratings, we modeled two extreme vignettes by predicting scores for a scenario in which all dimensions were set to their least favorable levels (negative vignette) and one in which all were set to their most favorable levels (positive vignette). Based on our regression model, the predicted comfort rating for the negative vignette was − 3.99, while the positive vignette received a predicted rating of 4.1 (on our scale from − 5 to + 5). The full text of these extreme vignettes can be found in Additional file: Tables S4 and S5.

### Subgroup analyses

For male participants, the dimensions remained significant, except for giving *recommendations without a reason* which did not influence comfort ratings (*β* = − 0.13, *p* = 0.18). For female participants, the *experience of the physician* only increased comfort if it was late career (*β* = 0.18, *p* < 0.05) and was no longer significant when being in mid-career (*β* = 0.08, *p* = 0.35). For female participants, the *sex of the patient* described in the vignette significantly influenced comfort ratings, as male patients were rated significantly lower compared to female patients (*β* = − 0.14, *p* < 0.05). The same was true for the *sex of the physician*, female participants rated male physicians as less comforting compared to female physicians (*β* = − 0.15, *p* < 0.05). The *R*^2^ for females (*R*^2^ = 0.31) was higher compared to that of the whole sample (*R*^2^ = 0.27) and the male subsample (*R*^2^ = 0.22).

Among the subgroup of healthcare workers (*N* = 324), the *experience of the physician* and the *relationship with the physician* did not influence comfort significantly. *Recommendations given without reason* were the only type of recommendations that remained significantly negative (*β* = − 0.38, *p* < 0.05). When looking at the sample and excluding people working in healthcare, we found no differences regarding the importance of each dimension which showed as the same dimensions being significant as in the whole sample.

The subsample of participants who had a chronic disease or who had someone close with a chronic disease showed that *experience of the physician* did not significantly influence comfort (*β* = − 0.07, *p* = 0.53; *β* = 0.16, *p* = 0.13). If *recommendations were given without reason*, they negatively influenced comfort (*β* = − 0.27, *p* < 0.05). In all subsamples, *clarity*, *taking time*, *continuity of care*, *self-disclosure*, and *expression of sadness* remained positively significant.

## Discussion

Our study showed that taking time, precise information delivery, expression of continuity of care, physicians’ self-disclosure, expression of sadness (including tearing up), and recommendations tailored to patient’s wishes are pivotal in increasing comfort with a physician during SICs from the public perspective. While factors such as taking time, precise information delivery, and expression of continuity of care count with good evidence [13, 18, 19, 25, 43–45], our study—through its national scale and factorial design—provides even more robust evidence for these aspects. It also underscores the importance of other communication styles and characteristics such as physicians’ self-disclosure of personal information, expression of sadness, and recommendations tailored to patient’s wishes, highlighting the need for more attention to their potential value in SICs. Furthermore, the significant difference in comfort ratings between our modeled extreme vignettes reinforces the overall impact of communication style. This is evident in the markedly higher comfort rating for the most favorable communication approach compared to the least favorable one, emphasizing the role of effective communication in shaping comfort during SIC.

Self-disclosure is still seen as controversial, mainly because it can be perceived as a boundary violation from a healthcare provider’s perspective [29, 46]. Nevertheless, our results underscore that self-disclosing personal information and expressing one’s sadness, which can be categorized as *rapport-building* self-disclosures [47], can lead to heightened comfort ratings. Crying and tearing up remain understudied even though studies have shown that nearly half of all physicians have cried at their workplace, and at least one quarter had cried in the presence of a patient during the 12 months prior to the survey [48]. In the study by Janssens et al. [48], medical interns and physicians expressed slightly negative attitudes toward crying, seeing it as unprofessional and a sign of weakness, thus showing how pervasive the historical prohibition of these behaviors is within medicine [49]. Crying was only seen as appropriate by physicians when it was about the patient’s situation [48], which holds true for our vignettes and might be why it led to increased comfort ratings in our study. Supporting this view, palliative care physicians, rather than seeing crying and the expression of emotions as unprofessional or negative, perceive it as beneficial to the physician–patient relationship [50], observations which had earlier been made by Siegel [51] and Rousseau [49] who argued that crying and expressing sadness are ways for physicians to show their vulnerability and humanity. However, further investigation is warranted; physicians’ expressions of sadness and crying deserve more attention in education and research [48, 52] especially since evidence highlights that suppression and inhibition of emotions, including the suppression of tears, might influence physician’s well-being and increase their risk for burnout [53–55].

In times of patient-centered care [56] and shared decision-making [57], it is unsurprising that recommendations based on patient preference increase participant comfort. These findings highlight the significance of physicians offering a recommendation that agrees with patients’ wishes [58] and underscore the importance of physicians actively listening to patient preferences and basing decisions on those preferences during SICs. Additionally, our results indicate that the failure to link recommendations to patient preferences can compromise the physician–patient relationship and diminish comfort with the physician. Some physicians refuse recommendations, believing it undermines patient autonomy [39]. However, our results suggest that giving no recommendation is only worse when a recommendation is provided without any accompanying reason. We suggest always basing recommendations on patient preferences. In cases where time constraints exist, it may be preferable to refrain from providing a recommendation rather than offering one hastily without justification.

In the whole sample, a prior relationship with and experience of the physician increased comfort ratings, which is in line with the study by Nauck and Jaspers [59], which found that physician experience contributes to patients’ trust. Our results show that even a brief prior relationship between the physician and the patient resulted in increased comfort ratings. Therefore, it seems advisable for physicians to introduce themselves in a prior meeting before conducting a SIC. However, in the subsample of healthcare workers in our study, these two factors did not influence comfort ratings. There has only been limited research on physicians and other healthcare professionals becoming patients, and one potential explanation could be due to the training and exposure of healthcare workers so that they prioritize other aspects of care over a prior relationship or may understand system constraints that do not allow for many choices. Additionally, healthcare workers may be placing a greater emphasis on competence and adherence to professional standards rather than the duration or nature of the individual physician–patient relationship. However, these assumptions require further investigation. Future research should also investigate the interaction between the investigated dimensions or the interaction between participant characteristics such as occupation in healthcare, age, or gender and the presented dimensions.

This study is not without limitations. First, the dimensions we investigated were selected based on the literature, and the described vignette can be seen as an oversimplification of the real world and SIC. It is important to note that factorial vignettes are inherently measuring imagined comfort, rather than actual comfort. However, the factorial survey provides experimentally manipulated scenarios, allowing insight into underlying mechanisms that contribute to comfort. Factorial surveys are increasing internal validity and allowing causal interpretation of dimension and outcome at the cost of external validity [60]. Second, while factorial survey methods have been used in healthcare communication research, our study relied on a general population sample rather than clinical patients. Participants were asked to imagine themselves as the patient in the described scenario, which introduces an additional layer of hypotheticality. Research supports the validity of analogue patient assessments of healthcare communication [32, 33], but it remains uncertain to what extent their perceptions align with those of actual patients experiencing serious illness conversations. This should be considered when interpreting our findings, and future studies could compare responses from analogue patients with those of clinical patients to assess potential differences in perspectives. Third, the potential simplification of SIC is reflected in the fact that our model explained 27.3% of the variance in comfort with the physician. While this may be seen as low, it is essential to consider that SICs are generally uncomfortable for patients and physicians alike [1, 3]. It may not be possible to make them entirely comfortable for patients. The score could also indicate that comfort with a physician depends on other aspects not included in our vignettes such as non-verbal behavior, race, or other sociodemographic characteristics. Future research could depict vignettes not as text but as video vignettes using actors to increase realism and external validity. Additionally, even though we applied quotas to achieve a representative sample regarding age, sex, and the three languages spoken in Switzerland, our sample consisted of a higher-than-expected amount of healthcare workers, and participants with higher education were also overrepresented. This could be due to self-selection bias and a higher interest of healthcare workers in research projects focusing on topics relevant to them. Lastly, the concept of patient comfort deserves more attention regarding its conceptualization in future research as there is no standardized and agreed-upon definition for this concept. Due to feasibility constraints, we relied on a single-item question to assess patient comfort rather than a comprehensive scale. Despite these limitations, our study has important strengths: the factorial survey approach allowed us to investigate several different dimensions simultaneously that are relevant during serious illness conversations using a large sample from the public, broadening the current knowledge within the field of SICs. Importantly, our results reflect perspectives from the general public, allowing us to contribute a broader view of societal attitudes toward SIC. Understanding public perceptions is valuable, as they may shape expectations and potentially influence how individuals experience SIC as future patients or family members.

## Conclusions

Our study highlights communication styles that physicians can use to enhance comfort during SICs and the characteristics of the physician patient relationship that can foster comfort. In line with previous research, we found that taking time, providing clear information, and ensuring continuity of care are pivotal in enhancing comfort, though our study provides an even more rigorous basis for those conclusions. Also relevant for increasing comfort levels are the physicians’ expression of sadness, the self-disclosure of personal information relevant to the consultation, and having a prior relationship with the patient. Exploring how to teach these communication styles and how to incorporate these behaviors and styles into medical training and in serious illness communication could lead to more valuable end-of-life discussions and decision-making.

Supplementary information.

Additional file 1: Table S1 Example of vignette. Table S2 Correlations across dimensions. Table S3 Coding of dimensions and levels. Table S4 Example of a negative vignette. Table S5 Example of a positive vignette.

## Supplementary Information

Supplementary Material 1.

## Data Availability

The datasets generated and analysed during the current study are not publicly available but are available from the corresponding author on reasonable request.

## Abbreviation

SIC(s): Serious illness conversation(s)
