# Supplementary material for: Comforting styles of serious illness conversations: a Swiss wide factorial survey study
Source: BMC Med. 2025 Apr 14;23:218. doi: 10.1186/s12916-025-04046-6 (PMC11995521; doi:10.1186/s12916-025-04046-6)
Supplement: Supplementary file 1 — Supplementary Material 1. [file 12916_2025_4046_MOESM1_ESM.docx]

**Additional file 1**

**Comforting styles of serious illness conversations: A Swiss wide factorial survey study**

Robert Staeck, Carsten Sauer, Steven Asch & Sofia C. Zambrano

Table S1 Example of vignette

Table S2 Correlations across dimensions

Table S3 Coding of Variables

Table S4. Example of a negative vignette

Table S5. Example of a positive vignette

**Table S1. Example of a vignette**

| Imagine observing an interaction between a **male** **35-year-old** patient and **a female** physician with **5 years of experience**. The patient **is meeting the physician for the first time**. In communicating that the patient has only about a year to live, the physician gives **only brief explanations of the disease and prognosis with complicated and technical language that the patient finds hard to understand**.  **After providing this information, the physician tells the patient that her own father had the same illness and understands how difficult facing this situation is**. After **explaining that she has a full schedule and is only able to address the most important questions the physician tells the patient that he should stop chemotherapy and begin palliative care**. **The physician expresses how sad it makes her to give these bad news**. Before the end of the consultation, the physician lets the patient know, **that she will remain available for the patient, in addition to the new team of physicians that will now be involved in providing medical care**. |
| --- |

*Note.* Levels marked in bold

**Table S2. Correlations across dimensions**

|  | Expe. | Sex Phy. | Sex Pat. | Age Pat. | Relat. | Clarity | Self. | Time | Rec. | Expr. | Cont. |
| --- | --- | --- | --- | --- | --- | --- | --- | --- | --- | --- | --- |
| Expe. | 1.00 |  |  |  |  |  |  |  |  |  |  |
| Sex Phy. | 0.00 | 1.00 |  |  |  |  |  |  |  |  |  |
| Sex Pat. | 0.01 | -0.01 | 1.00 |  |  |  |  |  |  |  |  |
| Age Pat. | 0.01 | 0.00 | 0.00 | 1.00 |  |  |  |  |  |  |  |
| Relat. | 0.00 | 0.01 | -0.01 | -0.01 | 1.00 |  |  |  |  |  |  |
| Clarity | 0.01 | 0.00 | -0.02 | 0.01 | 0.00 | 1.00 |  |  |  |  |  |
| Self. | 0.00 | 0.00 | 0.01 | 0.00 | 0.01 | 0.01 | 1.00 |  |  |  |  |
| Time | 0.00 | 0.01 | -0.01 | 0.01 | 0.02 | 0.00 | 0.00 | 1.00 |  |  |  |
| Rec. | 0.01 | -0.01 | 0.01 | 0.00 | 0.00 | 0.00 | -0.01 | 0.01 | 1.00 |  |  |
| Expr. | 0.01 | -0.01 | 0.00 | 0.00 | 0.00 | 0.00 | 0.00 | 0.01 | 0.00 | 1.00 |  |
| Cont. | -0.01 | -0.01 | -0.02 | 0.01 | 0.00 | -0.01 | 0.01 | 0.00 | 0.01 | 0.01 | 1.00 |

*Note*. Expe.: Experience of physician; Sex Phy.: Sex of physician; Sex Pat.: Sex of patient; Age Pat.: Age of patient; Relat.: Prior relationship to physician; Clarity: Clarity of information; Self.: Self-disclosure; Time: Physician takes time; Rec.: Recommendation; Expr.: Expression of Sadness; Cont.: Continuity of care.

**Table S3. Coding of Dimensions and Levels**

| Dimensions/Levels | Coding |
| --- | --- |
| Experience |  |
| Early career (Reference) | 0 |
| Mid career | 1 |
| Late career | 2 |
|  |  |
| Sex Physician |  |
| Female (Reference) | 0 |
| Male | 1 |
|  |  |
| Sex Patient (male) |  |
| Female (Reference) | 0 |
| Male | 1 |
|  |  |
| Age Patient |  |
| 35 years old (Reference) | 0 |
| 55 years old | 1 |
| 80 years old | 2 |
|  |  |
| Prior relationship |  |
| No (Reference) | 0 |
| Short | 1 |
| Long | 2 |
|  |  |
| Clarity of information (yes) |  |
| No (Reference) | 0 |
| Yes | 1 |
|  |  |
| Self disclosure |  |
| No (Reference) | 0 |
| Yes | 1 |
|  |  |
| Time (long) |  |
| Short (Reference) | 0 |
| Long | 1 |
|  |  |
| Recommendation |  |
| No (Reference) | 0 |
| Yes, without reason | 1 |
| Yes, based on experience | 2 |
| Yes, patient based on patient preference | 3 |
|  |  |
| Expression of sadness |  |
| No (Reference) | 0 |
| Yes, in words | 1 |
| Yes, in words and tears up | 2 |
|  |  |
| Continuity of care |  |
| No (Reference) | 0 |
| Yes | 1 |

**Table S4. Example of a negative vignette**

| Imagine observing an interaction between a **female 55-year-old** patient and **a male** physician with **5 years of experience**. The patient **is meeting the physician for the first time**. In communicating that the patient has only about a year to live, the physician gives **only brief explanations of the disease and prognosis with complicated and technical language that the patient finds hard to understand**.  After **explaining that he has a full schedule and is only able to address the most important questions the physician tells the patient that he should stop chemotherapy and begin palliative care**. Before the end of the consultation, the physician lets the patient know, **he will lose contact with the patient, because they will be referred to a different team of physicians.** |
| --- |

*Note.* Levels marked in bold; predicted comfort rating = -3.99 on a scale from -5 to +5

**Table S5. Example of a positive vignette**

| Imagine observing an interaction between a **male** **80-year-old** patient and **a female** physician with **5 years of experience**. The patient **has known the physician since this recent hospitalization**. In communicating that the patient has only about a year to live, the physician gives **detailed and clear explanations of the disease and prognosis that the patient seems to understand well.**  **After providing this information, the physician tells the patient that her own father had the same illness and understands how difficult facing this situation is**. After **taking** **enough time to listen to the patient and to answer questions the physician tells the patient that based on the wishes expressed by the patient during the conversation, his recommendation is to stop chemotherapy. The physician expresses how sad it makes her to give these bad news and tears up**. Before the end of the consultation, the physician lets the patient know, **she will remain available for the patient, despite the fact that a new team of physicians will now be involved in their care** |
| --- |

*Note.* Levels marked in bold; predicted comfort rating = +4.1 on a scale from -5 to +5
